# Increased Cell Detachment Ratio of Mesenchymal-Type Lung Cancer Cells on pH-Responsive Chitosan through the β3 Integrin

**DOI:** 10.3390/md17120659

**Published:** 2019-11-23

**Authors:** Chia-Hsiang Yen, Tai-Horng Young, Meng-Chi Hsieh, Li-Jen Liao, Tsung-Wei Huang

**Affiliations:** 1 Department of Biomedical Engineering, College of Medicine and College of Engineering, National Taiwan University, Taipei 10051, Taiwan; f04548042@ntu.edu.tw (C.-H.Y.); thyoung@ntu.edu.tw (T.-H.Y.); markhsieh814@gmail.com (M.-C.H.); 2 Department of Electrical Engineering, College of Electrical and Communication Engineering, Yuan Ze University, Taoyuan 32003, Taiwan; dtent87@gmail.com; 3 Department of Otolaryngology, Far Eastern Memorial Hospital, Taipei 220, Taiwan

**Keywords:** pH-responsive chitosan, lung cancer, epithelial-mesenchymal transition, transforming growth factor-β1, cell detachment ratio, integrins

## Abstract

Chitosan is sensitive to environmental pH values due to its electric property. This study investigates whether the pH-responsive chitosan assay can provide a simple method to evaluate the aggressive behavior of cancer cells with cell detachment ratio. The epithelial–mesenchymal transition (EMT) is induced with transforming growth factor-β1 (TGF-β1) in the human non-small cell lung cancer cell line (A549). EMT-induced cells and untreated cells are cultured on chitosan substrates at pH 6.99 for 24 h, followed by pH 7.65 for 1 h. The cell detachment ratio (CDR) on pH-responsive chitosan rises with an increasing of the TGF-β1 concentration. The protein array reveals that the expression levels of the α2, α3, α5, β2, and β3 integrins are higher in EMT-induced A549 cells than in untreated cells. A further inhibition assay shows that adding β3 integrin blocking antibodies significantly decreases the CDR of EMT-induced cells from 32.7 ± 5.7% to 17.8 ± 2.1%. The CDR of mesenchymal-type lung cancer cells increases on pH-responsive chitosan through the β3 integrin. Notably, the CDR can be theoretically predicted according to the individual CDR on the pH-responsive chitosan surface, irrespective of heterogeneous cell mixture. The pH-responsive chitosan assay serves as a simple in vitro model to investigate the aggressive behavior of lung cancer including the heterogeneous cell population.

## 1. Introduction

Cancer has become the top leading cause of death in recent years. Tumor metastasis is a major factor in the severity of cancer and seems to be triggered by a specific mechanism called the epithelial–mesenchymal transition (EMT). The EMT plays a significant role in tumor metastasis. Most cancer cells at the original tumor location are epithelial-type cells that are stable and immobile. Cells undergoing the EMT become mesenchymal-type circulating tumor cells, which are more aggressive and have a better migratory ability. The regulation of cell migration requires the formation of focal adhesion and intermediate adhesion strength with the underlying matrix [1]. Integrins are a family of transmembrane receptors comprising two subunits, α and β. Researchers have identified the 18α and 8β subunits in mammals, subunits which can form at least 24 different integrin heterodimers [2]. These receptors facilitate interactions between cells and the extracellular matrix (ECM), participate in cytoskeleton organization, and play significant roles in cell signaling [3]. The invasion and detachment of tumor cells from the primary site is a major step in the formation of metastasis [4]. During tumor metastasis, cancer cells must detach from the ECM before the invasion. There is increasing evidence that integrins are involved in regulating a diverse array of cellular functions that are essential to the initiation and progression of cancer. Research of cancer metastasis to date, including biology and specific therapies against metastasis, has generally adopted the in vivo animal model [5]. However, the metastatic process can be usefully dissected into single steps or groups of events using in vitro techniques. A chemoinvasion assay is a common in vitro tool to assess cancer invasion with reconstituted basement membranes [6]. Nevertheless, no in vitro tool yet exists to evaluate the detachment step of cancer metastasis. Therefore, developing a simple in vitro model to investigate cancer detachment is beneficial for cancer research.

Chitosan is a widely-used biomaterial derived from chitin, the main component of the shells of crab, shrimp, and other crustaceans. It is a linear polysaccharide with many amino groups, and it is formed by deacetylation—the removal of the acetyl groups of chitin [7,8]. Chitosan is also a cationic polyelectrolyte, with an isoelectric point of 7.4 [9], and is thus sensitive to environmental pH values. Our previous works have demonstrated that pH-responsive chitosan can control cell attachment and detachment without trypsin or other chemicals by affecting the ability of fibronectin adsorption [10]. pH-responsive chitosan can also be adopted to separate mixed cells based on the different cell detachment ratios (CDR) on chitosan [11]. Since pH-responsive chitosan can control cell attachment and detachment, limited research has been performed on whether these mesenchymal-type cancer cells are easily detached from the pH-responsive chitosan surface or on whether the CDR on pH-responsive chitosan can provide a simple method to evaluate the aggressive behavior of cancer cells. This study aims to investigate the detachment of mesenchymal-type lung cancer on pH-responsive chitosan, and it further elucidates the detachment mechanism.

## 2. Results

### 2.1. The Effect of Transforming Growth Factor-β1 (TGF-β1) on Cellular Morphology

**Figure 1:**
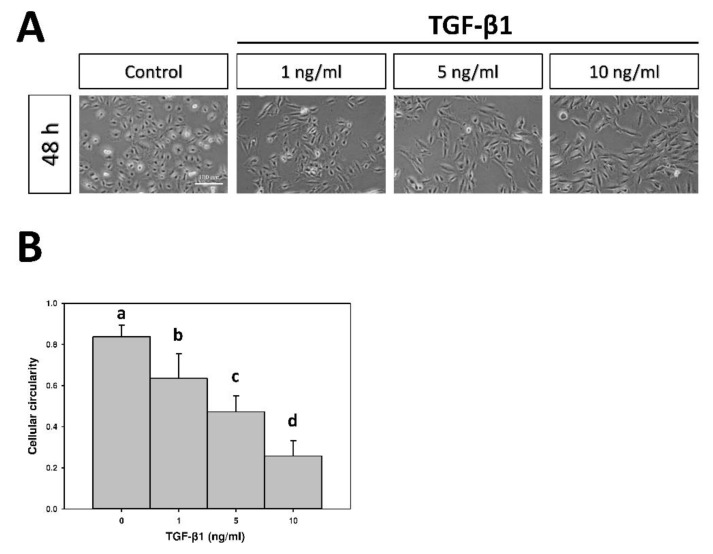
(**A**) Morphological alteration of A549 cells induced by transforming growth factor-β1 (TGF-β1) at different concentrations for 48 h on tissue culture polystyrene (TCPS). (**B**) The cellular circularity of A549 cells following TGF-β1 treatment. Data were calculated from four independent experiments (*n* = 4). Different letters indicate significant differences (*p* < 0.05). Original magnification: 40×. Scale bar = 100 μm.

Phase contrast microscopy revealed that untreated A549 cells, a cell line of human non-small cell lung cancer, on the tissue culture polystyrene (TCPS) had triangle-like forms and were mostly in aggregation in controls (Figure 1A). The A549 cells became more extended and elongated after being cultured in different concentrations of transforming growth factor-β1 (TGF-β1) for 48 h. Cells treated with 10 ng/mL of TGF-β1 had a very distinct morphology compared to the control group. These qualitative changes in cell morphology were confirmed by quantitation. An analysis of circularity described a biologically relevant feature as it mathematically measures the ratio of “area” to “perimeter,” normalized to one. The circularity of A549 cells in the control, 1 ng/mL, 5 ng/mL, and 10 ng/mL were 0.8 ± 0.1, 0.6 ± 0.1, 0.5 ± 0.1, and 0.3 ± 0.1, respectively (*p* < 0.05, Figure 1B). TGF-β1 treatment decreased cellular circularity, indicative of a larger deviation from a rounded shape.

### 2.2. Immunocytochemistry and Western Blots Analyses of EMT

**Figure 2:**
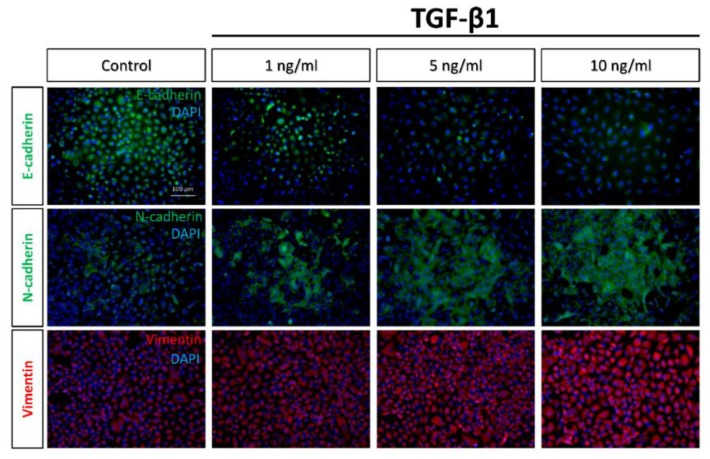
Immuno-staining of epithelial–mesenchymal transition (EMT) markers under different concentrations of TGF-β1 treatment in A549 cells. Micrographs show the epithelial marker E-cadherin (green) and mesenchymal markers N-cadherin (green) and, vimentin (red). Nuclei are counterstained with 4′,6-diamidino-2-phenylindole (DAPI) (blue). Original magnification: 200×. Scale bar = 100 μm.

**Figure 3:**
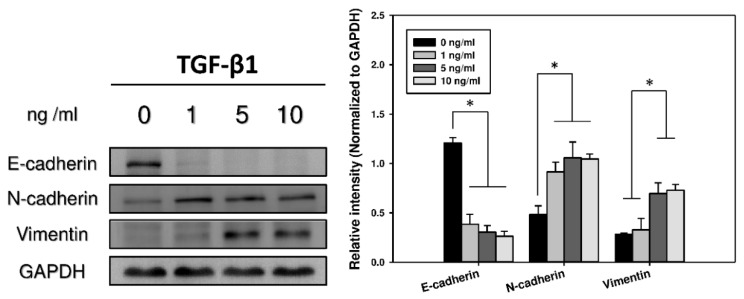
Immunoblotting of EMT-related protein expression under different concentrations of TGF-β1 treatment in A549 cells. Representative blots (left) show E-cadherin, N-cadherin, and vimentin protein levels. Densitometric analyses (right) were calculated from four independent experiments for protein levels normalized to glyceraldehyde 3-phosphate dehydrogenase (GAPDH) (*n* = 4). * *p* < 0.05 was considered significant.

Cadherins, a type of cell transmembrane adhesion glycoproteins that are dependent on calcium ions to function, mediate cell–cell adhesion through their extracellular domains; additionally, they connect to the actin cytoskeleton though its cytosolic tail. Typically, epithelial cells express E-cadherin, whereas mesenchymal cells express N-cadherin [12]. Furthermore, during the EMT process, the compositional change of cytoskeletal intermediate filaments initiates the expression of vimentin [13]. Thus, the development of the EMT was assessed by measuring the loss of E-cadherin as well as the acquisition of N-cadherin and vimentin [14]. Immunofluorescence revealed that the expression of E-cadherin fell as the concentration of TGF-β1 rose among these cells. In contrast, the expression of N-cadherin and vimentin increased as the concentration of TGF-β1 was raised (Figure 2). Western blot analyses further confirmed these results, which were consistent with the EMT phenomenon, in which the epithelial characteristics gradually disappeared and the properties of mesenchymal type emerged with time during the transition (Figure 3).

### 2.3. CDR of A549 Cells on pH-Responsive Chitosan

**Figure 4:**
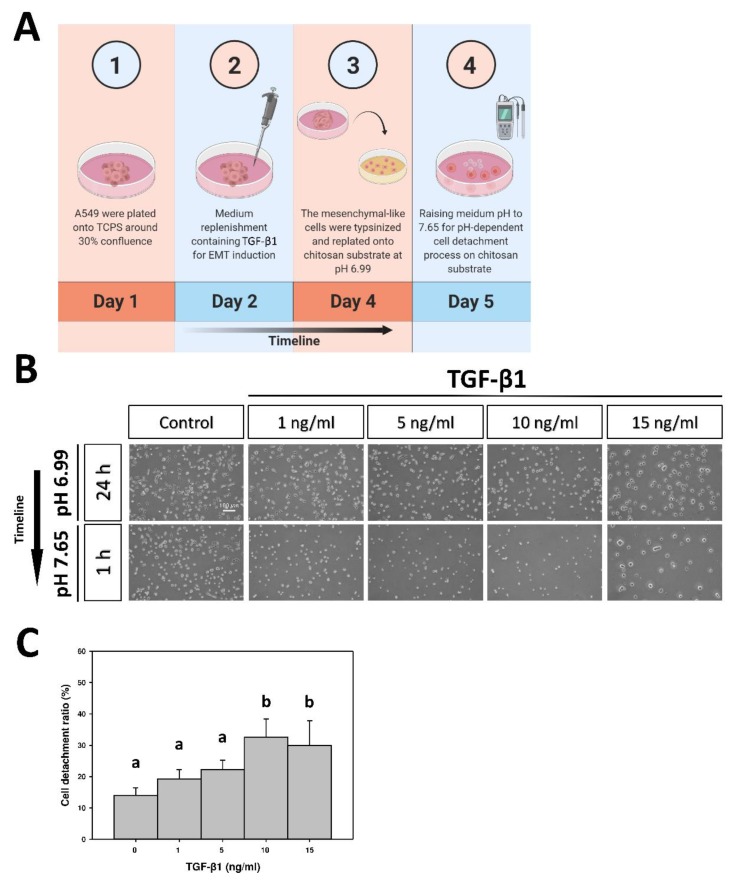
(**A**) Schematic depiction detailing the time course of cell detachment assay. (**B**) Morphologies of the TGF-β1-induced A549 cells subjected to the pH-dependent cell detachment process on chitosan. The TGF-β1-induced A549 cells were re-plated on a chitosan substrate at pH 6.99 for 24 h, and then the medium pH was raised to 7.65 for 1 h for cell detachment. (**C**) The cell detachment ratio of the TGF-β1-induced A549 cells on chitosan. Data were calculated from four independent experiments (*n* = 4). Different letters (a,b) indicate significant differences, while the same letter indicates no significant differences (*p* < 0.05). Original magnification: 40×. Scale bar = 100 μm.

**Figure 5:**
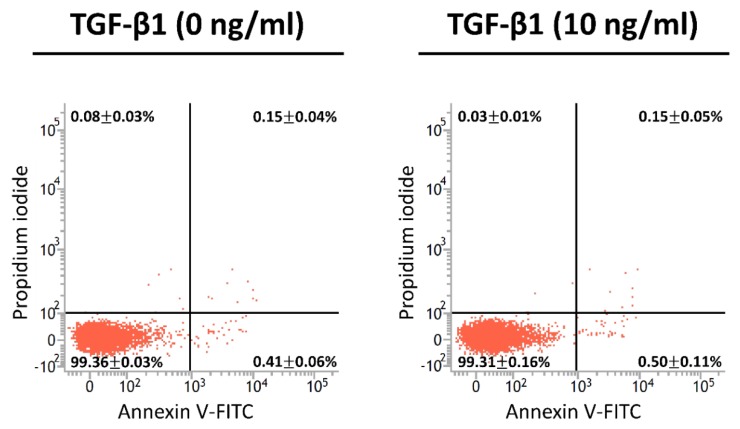
Cell apoptotic patterns of the detached population from the TGF-β1-induced (10 ng/mL) A549 cells and the untreated controls (0 ng/mL). Data were calculated from four independent experiments and expressed as mean ± standard deviation.

In the cell detachment assay, as illustrated by the time course (Figure 4A), A549 cells and EMT-induced cells were re-plated on chitosan at pH 6.99 for 24 h. Cells were detached from the chitosan substrate and counted after replacement by the pH 7.65 medium for 1 h. At pH 6.99, flattened, polygonal morphologies could be seen in the controls; when the EMT was induced, the polygonal shape became less obvious and exhibited a more rounded shape, as the concentration of TGF-β1 increased (Figure 4B). At pH 7.65, the spherical morphologies were observed in all conditions, indicating the slight attachment or ready-to-detach state of cells. In addition, as the concentration of TGF-β1 increased, the number of remaining adherent cells on chitosan substrate was reduced (Figure 4B). Quantitatively, the CDR was 14.1 ± 2.4% in untreated cells; increased as the concentration of TGF-β1 rose, and reached the plateau ratio of 32.7 ± 5.8% in EMT-induced cells with 10 ng/mL of TGF-β1 (Figure 4C). These results indicate that the cell detachment ability on pH-responsive chitosan rose with the increasing of the TGF-β1 concentration. The concentration of 10 ng/mL was adopted for further experiments. On the other hand, the apoptotic pattern of the detached population from the EMT-induced A549 cells and the untreated controls after cell detachment assay was examined by flow cytometric analyses. Annexin V was used as a probe to detect apoptotic cells by binding to phosphatidylserine upon the cell membrane. Propidium iodide, a small fluorescent molecule, is impermeant to live cells and apoptotic cells, but it stains dead cells [15,16]. The results revealed no early apoptosis in both A549 cells with and without TGF-β1 treatment (0.50 ± 0.11% and 0.41 ± 0.06%, respectively) (Figure 5). Thus, the cell detachment was due to the pH-dependent deprotonation of chitosan instead of cell death.

### 2.4. Expressions of Integrin in EMT-Induced A549 Cells and the Effect of Integrin Inhibitors on CDR

**Figure 6:**
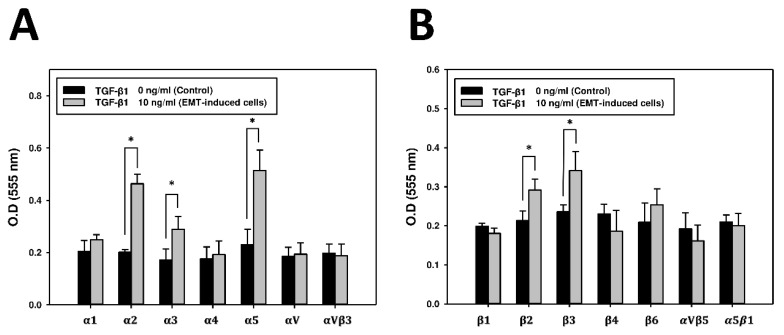
The expression of a subset of integrins in TGF-β1-induced (10 ng/mL) A549 cells, as compared to the untreated control group (0 ng/mL), was analyzed using the α/β-integrin array combo kit. (**A**) Results of the alpha integrin fluorometric array. Data are expressed as mean ± standard deviation of triplicate samples in each subunit. (**B**) Results of the beta integrin fluorometric array. Data are expressed as mean ± standard deviation of triplicate samples in each subunit. * *p* < 0.05 was considered significant.

**Figure 7:**
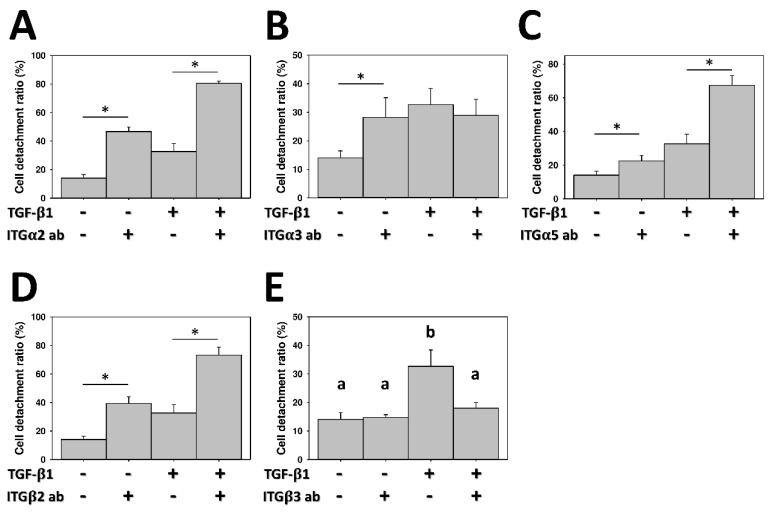
The effect of integrin blocking antibodies on the cell detachment ratio of lung cancer on pH-responsive chitosan. Specific neutralizing antibodies were added into the culture medium for blocking integrin-mediated cell detachment in TGF-β1-induced (10 ng/mL) A549 cells, as compared to the untreated control group (0 ng/mL). (**A**) α2 integrin; (**B**) α3 integrin; (**C**) α4 integrin; (**D**) β2 integrin; and (**E**) β3 integrin. Data were calculated from four independent experiments for each integrin (*n* = 4). Significant differences are indicated by asterisk, and different letters (a,b) indicate significant differences, while the same letter indicates no significant differences (*p* < 0.05).

**Figure 8:**
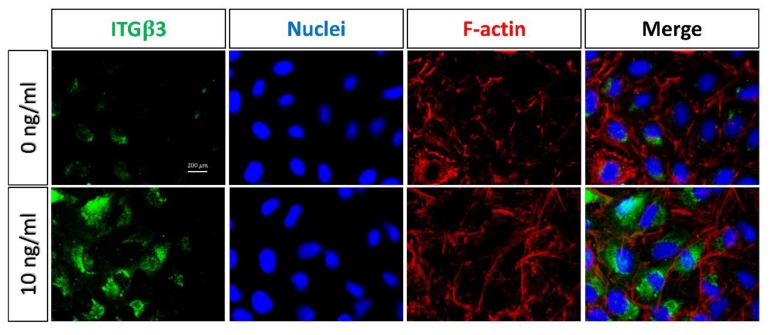
Immunofluorescent staining on TCPS. The expression of the β3 integrin (green) in TGF-β1-induced (10 ng/mL) A549 cells, as compared to the untreated control group (0 ng/mL). F-actin and nuclei were counterstained with phalloidin (red) and DAPI (blue), respectively. Original magnification: 400×. Scale bar = 200 μm.

Since integrins mediate cell migration and adhesion with interactions between cells and the extracellular matrix (ECM) [3], the expressions of integrins were analyzed with a protein array to elucidate the mechanism of regulating the CDR of A549 cells and EMT-induced A549 cells. The protein array revealed that the expression levels of the α2, α3, α5, β2, and β3 integrins were higher in EMT-induced A549 cells than in untreated cells (Figure 6). To determine the specific role of these integrins in the CDR, the relationship between integrins and the CDR of EMT-induced A549 cells was further evaluated with blocking antibodies. As shown in Figure 7, the CDR significantly increased in A549 cells treated with blocking antibodies of the α2, α3, α5, and β2 integrins, respectively. Simultaneously, adding blocking antibodies of the α2, α5 and β2 integrins also raised the CDR in TGF-β1-treated EMT-induced cells (Figure 7A,C,D). Conversely, the CDR was not significantly increased after treatment with β3 integrin blocking antibodies in A549 cells (Figure 7E). Notably, in the TGF-β1-treated cells, adding β3 integrin blocking antibodies significantly decreased the CDR from 32.7 ± 5.7% to 17.8 ± 2.1%, which was not significantly different from the level of the CDR of the untreated cells without blocking antibodies. (Figure 7E). Further immunofluorescence revealed that the expression level of the β3 integrin was higher in TGF-β1-treated cells than in controls (Figure 8).

### 2.5. CDR of Heterogeneous Cell Mixture on pH-Responsive Chitosan

**Figure 9:**
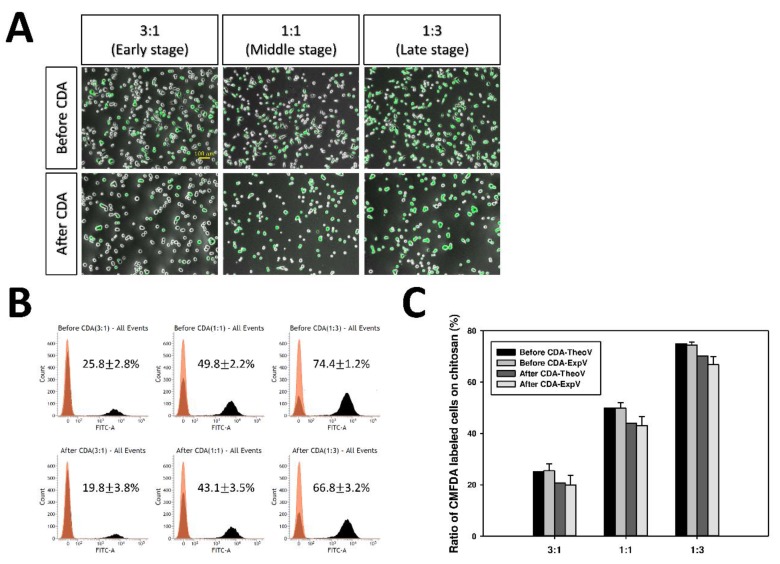
Cell detachment ratio of heterogeneous cell mixture on pH-responsive chitosan. The green 5-chloromethylfluorescein diacetate (CMFDA)-labeled- TGF-β1-induced (10 ng/mL) A549 cells and the untreated control group (0 ng/mL) were seeded on the chitosan substrate in different proportions. (**A**) Morphologies of different proportions of A549 cell mixture before and after a cell detachment assay (CDA). Original magnification: 50×. Scale bar = 100 μm. (**B**) The ratio of CMFDA-labeled-TGF-β1-induced cells on chitosan substrate was analyzed by flow cytometry and expressed as mean ± standard deviation from four independent experiments. (**C**) Bar graph shows the theoretical calculation value (TheoV) and experimental value (ExpV) of CMFDA-labeled-TGF-β1-induced cells on chitosan before and after CDA.

The initiation of metastasis during cancer progression requires invasion, which is enabled by the EMT. To simulate cancer heterogeneity, TGF-β1-induced EMT cells were regarded as aggressive metastatic cells. Therefore, a cell mixture of the green 5-chloromethylfluorescein diacetate (CMFDA)-labeled-TGF-β1-induced (10 ng/mL) cells and the untreated cells (0 ng/mL) was seeded on the chitosan substrate in different proportions (1:3, 1:1, and 3:1) to simulate the heterogeneous tumor population (Figure 9A). After a cell detachment assay with pH elevation, the percentage of green CMFDA-labeled-TGF-β1-induced cells on chitosan fell in all mixing proportions, indicating that TGF-β1-induced cells had a high tendency of cell detachment (Figure 9B). Moreover, the ratio of CMFDA-labeled-TGF-β1-induced cells on chitosan after pH-dependent cell detachment was nearly consistent with the theoretical calculation value (TheoV), regardless of the mixing proportion (Figure 9C). That is, the experimental value (ExpV) was predictable on chitosan using the calculation of the respective cell detachment ratio.

## 3. Discussion

EMT-induced circulating tumor cells flow into blood vessels and search for another place to survive, thus accomplishing cancer metastasis [17]. Therefore, this study adopted EMT-induced cancer cells to evaluate the CDR on pH-responsive chitosan. TGF-β1 is a key factor in the entire EMT course [18]. Many researchers have examined the EMT of human lung alveolar epithelial cell lines, A549, induced by TGF-β1 [19,20]. Some studies have reported the down-regulation of epithelial biomarkers, such as E-cadherin, as well as the up-regulation of mesenchymal markers such as vimentin and N-cadherin during the process of the EMT [17,18,21]. Cancer metastasis results in a loss of epithelial cell adhesion ability and increased mesenchymal invasive properties, enabling cancer cells to migrate to a secondary region. Cells not only become more aggressive and invasive after the EMT but also have advanced migratory ability. Several works have also indicated that the ability of cell migration increases as the EMT develops among many cancer cell varieties such as lung [22], prostate [23], and colon [24]. The results revealed a loss of E-cadherin, as well as the acquisition of N-cadherin and vimentin of A549, and this confirms that the EMT is induced in A549 cells with TGF-β1.

Cell adhesion is usually considered to be a multistep process involving the adsorption of ECM proteins such as fibronectin onto the substrate surface and the recognition of ECM components by cell surface receptors, followed by cytoskeletal rearrangements that lead to cell spreading [14,25]. Previous studies have demonstrated that a medium pH affects the ability of fibronectin adsorption on the chitosan surface [7]. Because the isoelectric point of chitosan is at pH = 7.4 [9], the chitosan surface becomes positively charged due to the protonation of the primary amine group when with a medium pH < 7.4, and lower medium pH leads to higher charge density on the chitosan surface. Since ECM proteins have a net negative charge at physiological pH [26], the increased fibronectin adsorption and adhesion of cells have been observed on chitosan surface at medium pH < 7.4. Conversely, cell adhesion is lower with a medium pH > 7.4 due to the reduced adsorption of the ECM proteins rejected by the deprotonated chitosan surface [10]. The CDR has been found to increase in proportion to the incubation time in pH 7.65 medium. However, a long incubation time has been shown to lead to increased cell death. Therefore, this study adopted an incubation time of 1 h in medium pH 7.65. Experimental results showed that the cell detachment was resulted from the pH-dependent deprotonation of chitosan instead of cell death (Figure 5). The CDR was 14% on pH-responsive chitosan without TGF-β1 treatment, and it increased to around 33% as A549 cells underwent the EMT. The CDR on pH-responsive chitosan thus rose parallel to the aggressiveness of lung cancer cells.

Different specific integrin signals allow for different cancer cells to detach from the neighboring cells, reorient their polarity during migration, and survive and proliferate in foreign microenvironments [27,28]. Integrins have diverse and dynamic expressions and functions during the EMT, as they can initiate and enforce the EMT and invasion. Some integrins provide adhesion between cells and the ECM, but others are upregulated to facilitate the cell migration and invasiveness of cancer cells through lamellipodia, filopodia, invadopodia and podosomes [29]. High levels of expression of many integrins are correlated with tumor progression and metastasis [30,31]. The β3 integrin has been shown to be often overexpressed in tumor cells, including lung cancer, melanoma, glioblastoma and breast cancer [32,33,34,35]. In this study, experimental results revealed that the α2, α3, α5, β2, and β3 integrins are upregulated in EMT-induced A549 cells compared with the original A549 cells. However, the CDR rose with the addition of blocking antibodies of the α2, α5, and β2 integrins, indicating that these integrins provide stable adhesions between EMT-induced A549 cells and chitosan and are not involved in regulating the CDR on chitosan. In contrast, the addition of β3 integrin blocking antibodies in EMT-induced A549 cells significantly lowers the CDR from 33% to 17%, which is not significantly different from that in untreated A549 cells (Figure 7E). This finding indicates that a high CDR of EMT-induced A549 cells on pH-responsive chitosan is mediated via the β3 integrin. pH-responsive chitosan can be used to evaluate the detachments of mesenchymal-type lung cancer cells by interacting with the β3 integrin.

The evolutionary processes during cancer progression require a source of heterogeneity within the cancer cell population. Highly metastatic clones from tumor-cell populations have been found to have a higher rate of genetic mutability than nonmetastatic clones from the same tumor [4]. Whether pH-sensitive chitosan can also be applied to measure the metastatic ability of the heterogeneous cancer cells was further investigated with a cell mixture of CMFDA-labeled-EMT-induced A549 cells and untreated cells. In these experiments, the CDR of mixture cells after pH-dependent cell detachment was nearly consistent with the theoretical calculation value, which was calculated using the respective cell detachment ratio. This result indicated that pH-responsive chitosan served as a model for investigating the detachment ability of cancer cells, including the heterogeneous cell population. However, the mechanism of cancer metastasis is complicated and is still not well explored. Whether this pH-responsive chitosan assay can be applied to evaluate other aggressive phenotypes of cancer cells requires further investigation.

## 4. Materials and Methods

### 4.1. Preparation of Chitosan Substrate

Chitosan (C3646, Sigma-Aldrich, St. Louis, MO, USA) was dissolved in 3% (v/v) acetic acid to prepare a 0.5% (w/v) chitosan solution and filtered out acid-insoluble impurities by a 5 μm Isopore^TM^ membrane (Merck Millipore, Burlington, MA, USA). One hundred microliters of a chitosan solution were cast per cm^2^ of TCPS (Corning, NY, USA) and dried at 60 °C overnight to form a thin film. The prepared substrates were then neutralized with 0.5 N sodium hydroxide (Sigma-Aldrich) for 15 min. After that, they were washed thoroughly with deionized water before being exposed to ultraviolet light for 1 h.

### 4.2. Medium Preparation and Cell Culture

Dulbecco’s Modified Eagle Medium (DMEM; Gibco^®^, Thermo Fisher Scientific, Waltham, MA, USA) powder dissolved in deionized water supplemented with 10% fetal bovine serum (FBS; Biological Industries, Cromwell, CT, USA) and 1% antibiotic-antimycotic (Gibco^®^). The pH of DMEM was adjusted by the quantity of sodium bicarbonate (Sigma-Aldrich) added into the medium solution, 600 mg/L for pH 6.99 and 3600 mg/L for pH 7.65. The model cell line used in this research was the human, non-small cell lung cancer cell line A549 (obtained from Bioresource Collection and Research Center; BCRC, Hsinchu, Taiwan). In addition, cells were seeded on the prepared substrates at an initial density about 3.20 × 10^4^ cells/well and incubated at 37 °C with 5% CO_2_. For stimulating the EMT, the cells were treated with TGF-β1 (R&D systems, Minneapolis, MN, USA) for a specific period. In brief, cells were plated onto TCPS around 30% confluence on day 1 and replenished with fresh culture medium containing 10 ng/mL TGF-β1 on day 2 for the subsequent 48 h incubation.

In the experiment of the CDR of the heterogeneous cell mixture, the indicated cells were pre-stained with 10 μM of CellTracker^TM^ Green CMFDA (Invitrogen, Thermo Fisher Scientific, Waltham, MA, USA) dye in the medium for 30 min at 37 °C, then artificially mixed with indicated cells in proper ratios. The mixed cells were plated onto chitosan substrate for 24 h and followed by the pH-dependent detachment process. The ratio of the CMFDA-labeled cells was counted by flow cytometry (FACSVerse^TM^; BD Biosciences, Franklin Lakes, NJ, USA) before and after the cell detachment process. The theoretical value of cell ratio remaining on chitosan substrate after the medium pH elevation was calculated by Equation (1):

(1) $$\mathrm{Theoretical}\;\mathrm{Value}\;(\%)=\frac{{\left[A\right]}_{int}\times \left(1-r_{A,t}\right)}{{\left[A\right]}_{int}\times \left(1-r_{A,t}\right)+{\left[B\right]}_{int}\times \left(1-r_{B,t}\right)}\times 100\%$$

where “Theoretical Value” indicates the theoretical calculation value of the type A cell remaining on the chitosan substrate after the medium pH elevation. “${\left[A\right]}_{int}$” and “${\left[B\right]}_{int}$” denote the initial cell seeding ratio of type A and type B cells on chitosan, respectively. “$r_{A,t}$” and “$r_{B,t}$” indicate the respective cell detachment ratio of type A and type B cells on the chitosan substrate after experiencing pH-dependent detachment process for the indicated time (t).

### 4.3. Morphometric Measurement

Morphometric analyses were modified from the previous study [36]. The representative images taken by a phase contrast microscope (Olympus, Tokyo, Japan) were measured using ImageJ software (National Institutes of Health, USA). The cellular circularity (σ), a dimensionless quantity that numerically describe the shape of an object, is defined as Equation (2):

(2) $$\sigma =\frac{4\pi A}{P^{2}}$$

where A is the apparent surface area of a cell and P is the cell perimeter. The value of the cellular circularity is 1 for a perfectly round cell and approaches 0 for an elongated cell.

### 4.4. Immunocytochemical Staining

Cells were first fixed with 4% paraformaldehyde and permeabilized with 0.1% Triton X-100 (Sigma-Aldrich). Next, after being blocked in 3% bovine serum albumin (BSA; HyCell, Taipei, Taiwan) for 30 min at room temperature, the samples were then incubated with the following antibodies in respective dilutions overnight at 4 °C: anti-E-cadherin (610182; BD Biosciences), anti-N-cadherin (610921; BD Biosciences), anti-vimentin (ab45939; Abcam, Cambridge, UK), and anti-β3 integrin (MAB1957; Merck Millipore). After phosphate-buffered saline (PBS) washing, samples were incubated with the Cy3-conjugated goat anti-rabbit IgG/Alexa Fluor 488-conjugated goat anti-mouse IgG (Jackson ImmunoResearch Laboratories, West Grove, PA, USA) and counterstained with 4′,6-diamidino-2-phenylindole (DAPI; Santa Cruz Biotechnology) for 60 min at room temperature in the dark. These immuno-stained samples were captured using a fluorescent microscope (Leica, Wetzlar, Germany).

### 4.5. Immunoblotting

The protein expression was quantified by a western blot analysis. In brief, the harvested cells were lysed by a radio-immunoprecipitation assay buffer (RIPA; Roche, Basel, Switzerland) containing protease inhibitors, then resolved by 10% sodium dodecyl sulfate-polyacrylamide gel electrophoresis (SDS-PAGE), and transferred onto 0.45 μm polyvinylidene fluoride membranes (Millipore). The immunoblots were subsequently blocked in 5% BSA and probed with primary antibodies: anti-E-cadherin (ab15148; Abcam), anti-N-cadherin (ab76011; Abcam), anti-vimentin (ab45939; Abcam), and anti-glyceraldehyde 3-phosphate dehydrogenase (GAPDH; ab181603; Abcam) at 4 °C overnight. After extensive washing by TBST (Tris-buffered saline, 0.1% Tween 20), the immunoblots were incubated with horseradish peroxide-conjugated secondary antibodies (HRP-conjugated 2nd antibodies) and goat anti-rabbit IgG (ab6721; Abcam) for 1 h at room temperature, then developed using an Immobilon Western Chemiluminescent HRP Substrate (Merck Millipore). Immuno-reactive blots were detected by the UVP BioSpectrum 810 imaging system (Thermo Fisher Scientific). The protein expression levels were normalized to GAPDH as an internal loading control.

### 4.6. Cell Detachment Assay and Cell Detachment Ratio

The TGF-β1-treated cells trypsinized from TCPS were cultured on the chitosan substrates at pH 6.99 for 24 h, and this was followed by the changing medium pH to 7.65 for 1 h. The number of both detached and the remaining adherent cells on substrates were determined by the CyQUANT^®^ assay kit (Invitrogen) according to the manufacturer’s instructions. The cell detachment ratio was calculated by the following Equation (3):

(3) $$\mathrm{Cell}\;\mathrm{detachment}\;\mathrm{ratio}\;(\%)=\left(\frac{X}{X+Y}\right)\times 100\%$$

where *X* is the number of cells detached and *Y* is the number of cells remaining on the culture substrate.

### 4.7. Detection of Cell Apoptosis

The exposure of phosphatidylserine was determined by a flow cytometric annexin V/propidium iodide cell apoptosis kit, according to the manufacturer’s protocol. In brief, the detached cells were harvested, washed twice with cold PBS, and resuspended in an annexin-binding buffer at a density of 10^6^ cells/mL. An FITC (Fluorescein isothiocyanate)-annexin V (5 μL) and propidium iodide (1 μL) working solution were added to each 100 μL of the cell suspension containing 10^5^ cells. After 15 min of incubation in the dark, 400 μL of annexin-binding buffer were added, and samples were subsequently analyzed by flow cytometry by using FACSVerse^TM^ (BD Biosciences) and BD FACSuite software (BD Biosciences).

### 4.8. Integrin Protein Array

The α/β-Integrin-Mediated Cell Adhesion Array Combo Kit (ECM532; Merck Millipore) was used according to the manufacturer’s instructions. In brief, about 0.5 × 10^5^ of cells were seeded onto the substrates that immobilized with anti-α or anti-β integrins, and this seeding was incubated for 2 h. After being gently washed by an assay buffer, cells were stained with a cell stain solution. Finally, the extraction buffer was added to each well and left on an orbital shaker for 5–10 min at room temperature. The absorbance was determined at 555 nm on a microplate reader (SpectraMax M2e; Molecular Devices, San Jose, CA, USA).

### 4.9. Integrin-Mediated Functional Inhibition

The functional inhibition assay was performed by the antibody blockade method. During the pH adjustment process in the cell detachment assay, 5 μg/mL of specific neutralizing antibodies were added into the culture medium and incubated for an indicated period. The neutralizing antibodies used were as follows: anti-α2β1 integrin (ab24697; Abcam), anti-α3 integrin (MAB1952Z; Merk Millipore), anti-α5 integrin (ab78614; Abcam), anti-β2 integrin (MAB1962; Merk Millipore), and anti-β3 integrin (MAB1957; Merk Millipore).

### 4.10. Statistical Analysis

All data are presented as the mean ± standard deviation (Mean ± S.D.) of at least four independent experiments with three technical replicates. Statistical significance was calculated using a repeated measures analysis of variance (ANOVA) followed by Tukey’s post-hoc test (*p* < 0.05 was considered significant).

## 5. Conclusions

Experimental results demonstrate that the cell detachment ratio of mesenchymal-type lung cancer cells rises on pH-responsive chitosan through the β3 in vitro. The pH-responsive chitosan assay can act as a simple in vitro model to investigate the aggressive behavior of lung cancer cells including the heterogeneous cell population.
